# Vector competence of *Ixodes ricinus* instars for the transmission of *Borrelia burgdorferi* sensu lato in different small mammalian hosts

**DOI:** 10.1186/s13071-023-06110-7

**Published:** 2024-01-18

**Authors:** Lars K. Lindsø, Hildegunn Viljugrein, Atle Mysterud

**Affiliations:** 1 Centre for Ecological and Evolutionary Synthesis (CEES), Department of Biosciences, University of Oslo, Blindern, P.O. Box 1066, NO-0316 Oslo, Norway; 2 Norwegian Veterinary Institute, P.O. Box 64, NO-1431 Ås, Norway; 3 Norwegian Institute for Nature Research (NINA), Torgarden, P.O. Box 5685, NO-7485 Trondheim, Norway

**Keywords:** Vector competence, *Ixodes ricinus*, Lyme disease, Small mammals, Ticks

## Abstract

**Background:**

Many pathogens and parasites can infect multiple host species, and the competence of different hosts as pathogen reservoirs is key to understanding their epidemiology. Small mammals are important hosts for the instar stages of *Ixodes ricinus* ticks, the principal vector of Lyme disease in Europe. Small mammals also act as reservoirs of *Borrelia afzelii*, the most common genospecies of the *Borrelia burgdorferi* sensu lato (s.l.) spirochetes causing Lyme disease in Europe. However, we lack quantitative estimates on whether different small mammal species are equally suitable hosts for feeding* I. ricinus* and whether they show differences in pathogen transmission from host to tick.

**Methods:**

Here, we analysed the feeding success and prevalence of *B. burgdorferi* s.l. infections in 12,987 instar I. ricinus found on captured small mammals with known infection status in Norway (2018–2022).

**Results:**

We found that larvae were more likely to acquire a blood meal from common shrews (*Sorex araneus*, 46%) compared to bank voles (*Myodes glareolus*, 31%) and wood mice (*Apodemus sylvaticus*, 36%). Nymphs tended to be more likely to acquire a blood meal from wood mice (66%) compared to bank voles (54%). Common shrews harboured few nymphs (n=19). Furthermore, we found that larvae feeding on infected bank voles (11%) were more likely to be infected with *B. burgdorferi* s.l. than larvae on infected common shrews (7%) or wood mice (4%).

**Conclusions:**

Our study provides quantitative evidence of differences in suitability for the instar stages of *I. ricinus* across taxa of small mammals and highlights how even known small mammal host species can differ in their ability to feed ticks and infect larval ticks with the pathogen causing Lyme disease.

**Graphical Abstract:**

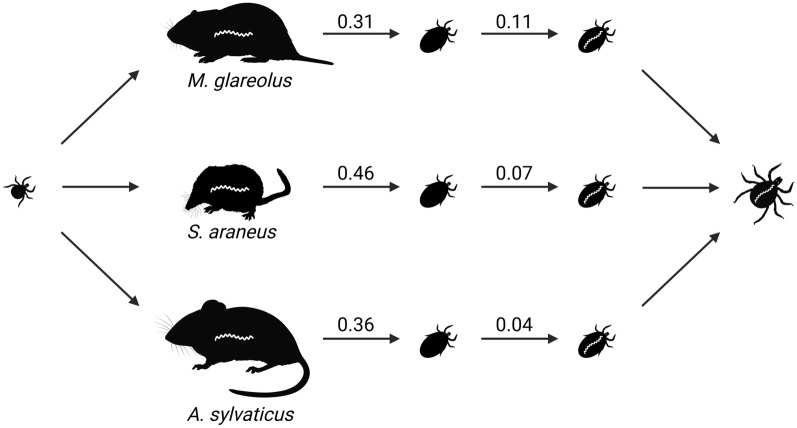

**Supplementary Information:**

The online version contains supplementary material available at 10.1186/s13071-023-06110-7.

## Background

Many emerging infectious diseases are vector-borne [1], where disease transmission is facilitated by vectors that carry and transmit pathogens from one host to another. In many host-vector-pathogen systems, the vector feeds on multiple host species [2]. Different host species may show variation in suitability to the vector itself [3, 4] and in host reservoir competence and immune response to vector-borne pathogens [5–7]. Consequently, different species may play different quantitative roles in maintaining vector populations and vector-borne pathogen reservoirs [2]. Understanding the role of different host species involved in the enzootic cycle of vector-borne pathogens is crucial for understanding their emergence and for predicting future development and disease risk.

Lyme disease is the most common vector-borne disease in North America and Europe [8]. The disease is caused by specific genospecies of spirochaetes within the *Borrelia burgdorferi* sensu lato (s.l.) complex [9] and is vectored by generalist species of ectoparasitic *Ixodes* spp. ticks [9]. Essential to the circulation of the pathogen are reservoir-competent vertebrate hosts that first become infected from an infectious tick bite and later infect new feeding ticks [10]. The main vector of *B. burgdorferi* s.l. in Europe, *Ixodes ricinus* [11], feeds on progressively larger vertebrate hosts through its stages as larva, nymph, and adult [12]. A blood meal is required for larvae and nymphs to commence moulting and develop to the next stage and for adult females to produce eggs [12]. Transovarial transmission of *B. burgdorferi* s.l. to hatched *I. ricinus* larvae is very rare [13, 14]. Rather, larvae imbibe the pathogen when feeding on a reservoir competent host [15, 16] and retain the pathogen through moulting so that they can subsequently infect new hosts as nymphs [12].

**Fig. 1:**
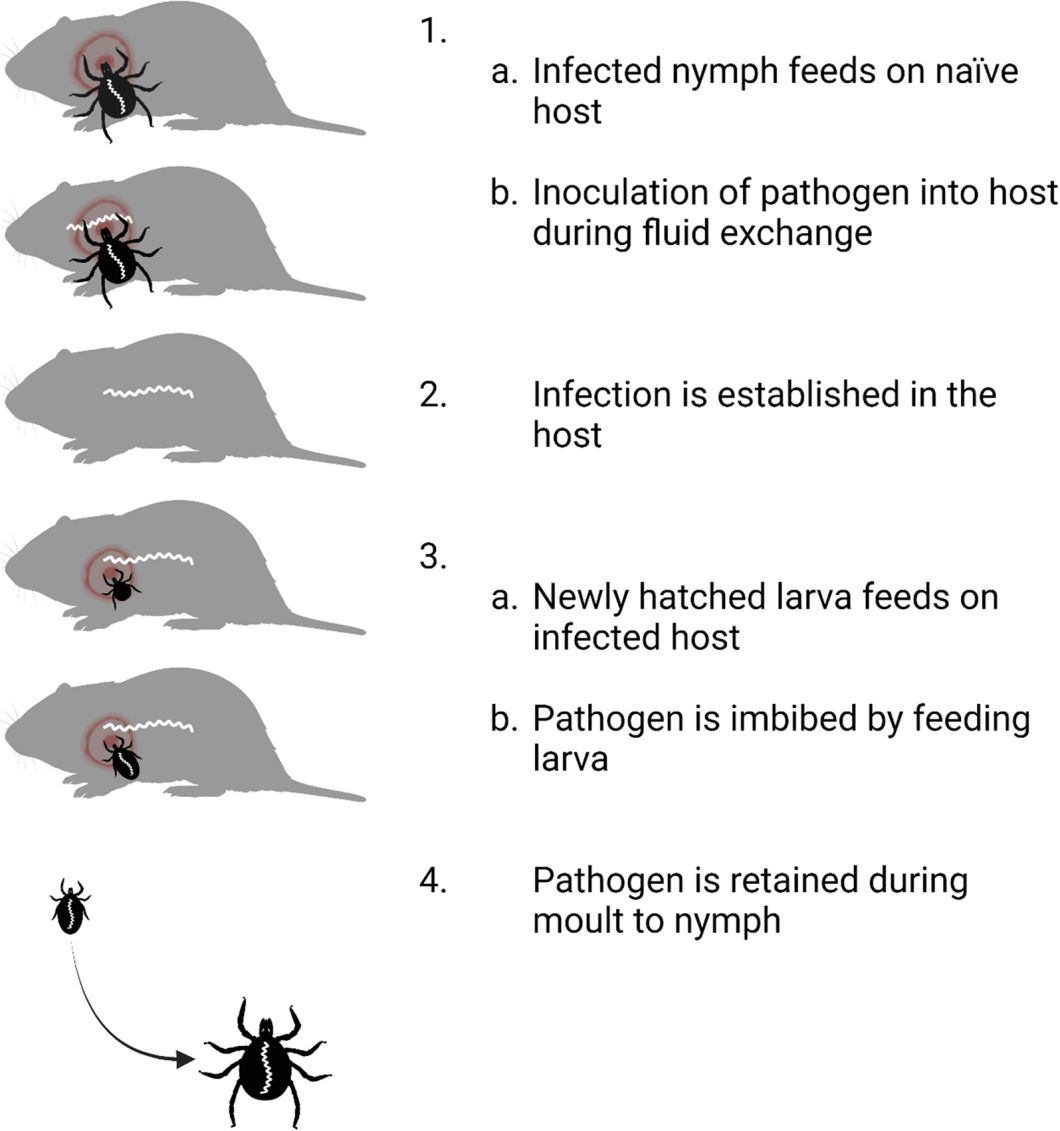
The successful transmission cycle of *Borrelia burgdorferi* s.l. requires both a competent vertebrate reservoir host and a competent *Ixodes* spp. tick vector. **1a** An infected nymph feeds on a naïve host. **1b** During fluid exchange between the nymph and the host, the pathogen is inoculated in the host. **2** The pathogen disseminates in host tissues and establishes a lasting infection. **3a** A newly hatched larva feeds on the infected host and **3b** imbibes the pathogen. **4** After a full blood meal, the infected larva detaches from the host and moults into an infected nymph. Created with BioRender.com

A large proportion of *I. ricinus* larvae feed on small mammals [17–19] that also act as reservoirs of the most common genospecies of *B. burgdorferi* s.l. in Europe: *Borrelia afzelii* [10, 20]. Small mammals are therefore a particularly important group of hosts, with their dual role in feeding larvae and reservoir competence producing infected nymphs [10, 18–20], i.e. the primary factor for Lyme disease hazard in humans [21]. However, the suitability of hosts for both feeding ticks and *B. burgdorferi* s.l. may differ across taxa [4, 7]. For example, ungulates are the main group of reproduction hosts that feed adult *I. ricinus* females but are not reservoir competent hosts for *B. burgdorferi* s.l. [22, 23]. Other vertebrate species may be reservoir competent for the pathogen but act as 'ecological traps' for ticks by killing attached ticks through grooming [24] or immune defences [25–29]. Indeed, only hosts on which larval ticks are able to (i) successfully ingest a blood meal and (ii) simultaneously imbibe the pathogen will moult into infected nymphs able to infect new hosts (Fig. 1). However, we lack quantitative estimates on whether small mammals across different taxa contribute equally to providing larvae with blood meals and to transmitting pathogen to feeding ticks.

In the present study, we analysed two principal components of *I. ricinus* vector competence on different small mammals captured over 5 years (2018–2022) in southeast Norway. We aimed to determine whether larval ticks were equally able to obtain a blood meal across small mammal species (Fig. 1 step 3a) and whether feeding larvae and nymphs are equally able to imbibe the pathogen from infected hosts across small mammal species (Fig. 1 step 3b). First, we analysed the number of successfully fed *I. ricinus* larvae and nymphs in a sample of 12,989 individual ticks from 498 captured individuals of different small mammal species (2018–2022). Second, we analysed infection prevalence of *B. burgdorferi* s.l. in 781 *I. ricinus* larvae from 58 captured small mammals (2018–2020) with positive *B. burgdorferi* s.l. infections.

## Methods

### Study area

The sampling was carried out in Vestby municipality, Viken, southeast Norway (59° 31′ 25.608''N 10° 41′ 13.884''E). The area is located in the boreonemoral vegetation zone [30] and consists of managed mixed forests, agricultural fields, and small settlements. Forests are mixed with Norway spruce (*Picea abies*), Scots pine (*Pinus sylvestris*), birch (*Betula* spp.), sessile oak (*Quercus petraea*), and Scots elm (*Ulmus glabra*). The forest understory typically consists of graminids (Gramineae), peat mosses (*Sphagnum* spp.), bilberry (*Vaccinium myrtillus*), and heather (*Calluna vulgaris*). Small mammalian hosts for *I. ricinus* ticks in the area include bank vole (*Myodes glareolus*), field vole (*Microtus agrestis*), wood mouse (*Apodemus sylvaticus*), common shrew (*Sorex araneus*), and pygmy shrew (*Sorex minutus*) [31]. Red squirrel (*Sciurus vulgaris*) is the most common medium-sized host for ticks [29], and roe deer (*Capreolus capreolus*) dominate as the main reproduction host to adult ticks [29].

### Small mammal trapping

We captured small mammals every spring and fall from 2018 to 2022. We had 25 trapping sites with four trap stations at each site, using a combination of lethal and live traps. All trap stations were deployed for 3 consecutive trap days with little to no precipitation within the period May 18 to 30 in spring and August 22 to 30 in fall. The four trap stations at each site consisted of either one live cage trap (Ugglan Special No. 3, Grahnab AB) or three common snap traps (to avoid trap saturation) and were spaced out in a 15 × 15-m square formation in accordance with the small quadrate method [32]. The proportion of live and lethal traps changed over the study period (Additional file 1: Table S1). The traps were checked once per day and all animals found alive were culled using cervical dislocation. All captured animals were killed to allow full tick counts on each individual and tissue sampling for later pathogen detection. The captured animals were stored in individual plastic bags in a freezer at −20 °C for further examination in the laboratory.

### Physical laboratory examinations

**Fig. 2:**
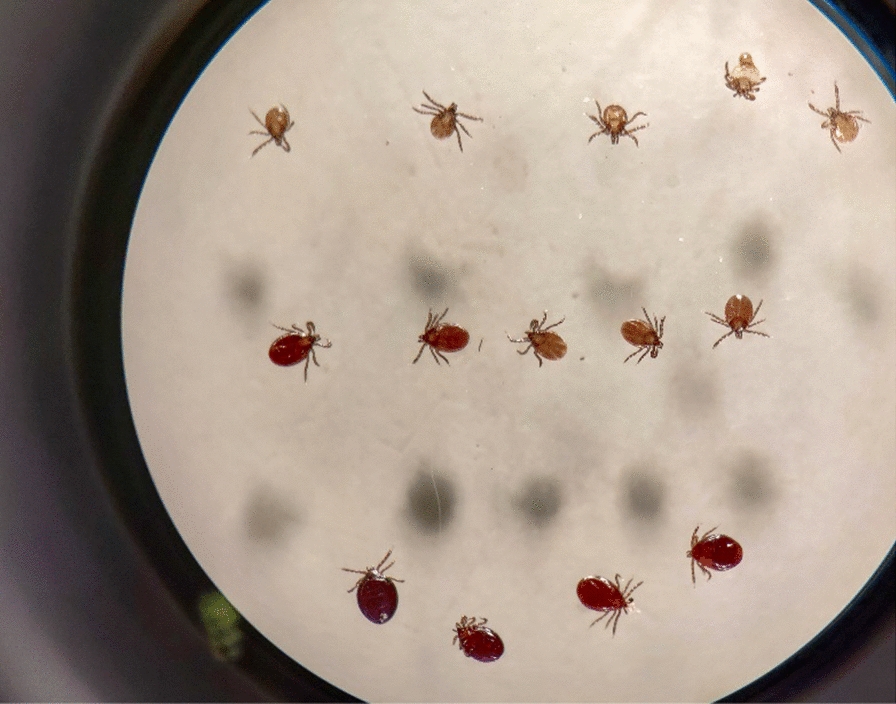
*Ixodes ricinus* larvae at different feeding stages. From top to bottom: unfed, partially engorged, and fully engorged *I. ricinus* larvae. Photo: Lars K. Lindsø

Tick examination on all captured small mammals was performed using a magnifying glass, tweezers, and a 20-min standardized examination time [19, 33]. During the examination, all ticks were placed aside before being counted. Ticks found in the respective individual plastic bags were also counted. The developmental and feeding stages of all individual ticks were determined under a stereomicroscope. The feeding stage of ticks was defined as a categorical trait: unfed, partially engorged, or fully engorged (Fig. 2). Ticks from 2020 to 2022 were also determined to species based on morphological characteristics [34], of which we verified using molecular methods as described below. Lastly, all small mammals were determined to species based on morphology by an expert on the species group (Jeroen van der Kooij, Nature communication, impact assessment and research Jeroen van der Koiij), and an ear tissue sample was collected from each host for pathogen detection.

### Genetic analyses

We used an established real-time multiplex quantitative PCR (qPCR) protocol [35] to determine the presence of *B. burgdorferi* s.l. in the ear tissue from small mammals. The qPCR protocol was implemented in our laboratory at the Centre for Ecological and Evolutionary Synthesis (CEES) at the University of Oslo (cfr. Additional file 1: Table S2) [31]. We have previously presented evidence that all *B. burgdorferi* s.l. sequences from small mammals in our system are *B. afzelii* [22]. To identify ticks from 2018 to 2019 to species and to verify morphological species determination of ticks from 2020 to 2022, we used a novel multiplex qPCR assay for identification of *Ixodes ricinus* and *I. trianguliceps* (unpubl., cfr. Additional file 1: Table S2) [31]. All qPCR results were analysed and given infection status or determined to species, respectively, in the application LightCycler^®^ 96 version 1.1.9.1320.

### Statistical analyses

Statistical analyses were conducted in R version 4.2.1 [36]. We analysed both the probability of successful feeding in *I. ricinus* larvae and nymphs (partially or fully engorged, Fig. 2) and the probability of *B. burgdorferi* s.l. infections in *I. ricinus* larvae from positive hosts using mixed effects logistic regression models with the package glmmTMB version 1.8.1 [37]. *Ixodes trianguliceps* was not considered because of paucity of data and their limited impact on the *B. burgdorferi* transmission cycle [38, 39]. The models were fitted jointly for the most abundant host species (bank vole, common shrew, and wood mouse). The nymphal feeding model included only ticks from bank voles and wood mice because of a paucity of nymphs on common shrews (Table 1). First, we tested whether season (spring or fall) influenced tick feeding or tick infection probability in separate models with season as a fixed factor variable and trap station, year, and host ID as random intercept. The random intercepts included trap station and year to account for spatial and temporal autocorrelations, respectively, and host ID to account for individual heterogeneities in host susceptibility to tick infestation and infection [31]. Second, we built separate models on larval feeding, nymphal feeding, and probability of *B. burgdorferi* s.l. infections in larvae that included host species as a factor variable and host ID as random intercept. The tick larval and nymphal feeding models also included whether the host was found dead or alive as a factor variable to control for potential confounding effects and trapping session for each year and season (10 levels) as random intercept. The infection model also included fed status (fed or unfed) as a factor variable and trap station as random intercept. We used the package DHARMa version 0.4.6 [40] to inspect residuals and assess the goodness of fit for each model and the package ggeffects version 1.2.3 [41] to generate back-transformed model estimates and confidence intervals.

**Table 1:** Number of fed and unfed *Ixodes ricinus* larvae and nymphs from captured small mammals in southeastern Norway (2018–2022)

	Hosts		*I. ricinus* larvae			*I. ricinus* nymphs		
	Live	Sum	Fed	Unfed	Sum	Fed	Unfed	Sum
*Apodemus sylvaticus*	71 (49%)	144	1292	3148	4440	174	119	293
*Microtus agrestis*	9 (69%)	13	78	173	251	30	9	39
*Myodes glareolus*	92 (38%)	241	1586	4299	5885	95	113	208
*Sorex araneus*	4 (5%)	85	550	1172	1722	8	11	19
*Sorex minutus*	0	15	35	95	130	0	0	0
Sum	176 (35%)	498	3541	8887	12,428	307	252	559

Finally, we calculated the mean number of fed and infected larvae produced by each host species (${\text{FI}}_{{l}_{i}}$) as a function of host infection prevalence, mean larval tick burden, estimated probability of successfully fed larvae, and estimated probability of *B. burgdorferi* s.l. infections in fed larvae:

$${\text{FI}}_{{l}_{i}}= {{n}_{i}\times Bb}_{i}\times {T}_{{l}_{i}}\times {F}_{{l}_{i}}{\times (Bb}_{{l}_{i}}|{F}_{{l}_{i}})$$

where ${n}_{i}$ is the abundance of host species $i$, ${Bb}_{i}$ is the proportion of infected reservoir hosts in species $i$, ${T}_{{l}_{i}}$ is the population mean number of larval ticks on individual hosts, ${F}_{{l}_{i}}$ is the estimated proportion of fed larvae on a live host, and ${Bb}_{{l}_{i}}|{F}_{{l}_{i}}$ is the estimated proportion of infected larvae given successful feeding on a live host.

We calculated the mean number of blood-fed and infected *I. ricinus* larvae (${\text{FI}}_{{l}_{i}}$) assuming equal host abundance for each species (${n}_{i}$ = 100). We used bootstrap sampling (*n* = 1000) for the median number of *I. ricinus* larvae across captured individuals of each host species (${F}_{{l}_{i}}$). We randomly drew 1000 samples for the remaining variables using their respective standard errors as variation parameters, assuming a normal distribution. We calculated the proportion and standard error of hosts per species with *B. burgdorferi* s.l. infections (${Bb}_{i}$) from the raw data. We used the model estimated mean and standard error (drawing samples on the logit-scale of the model and back transforming to proportions) in both the proportion of fed larvae (${F}_{{l}_{i}}$) and proportion of infected larvae given successful feeding (${Bb}_{{l}_{i}}|{F}_{{l}_{i}}$). We calculated the mean product across the 1000 samples and the interquartile range.

## Results

In total, we captured 557 small mammalian hosts, of which 258 (46%) were bank voles, 156 (28%) wood mice, 106 (19%) common shrews, 23 (4%) pygmy shrews, and 14 (3%) field voles. The number of captured individuals per species, season, and year is detailed in the Supporting information (Additional file 1: Table S1). Common shrews were most often infected with *B. burgdorferi* s.l. (54%, SE = 0.05), followed by bank voles (38%, SE = 0.03) and wood mice (35%, SE = 0.04; Additional file 1: Table S3). The determination of tick species by morphology was consistent with the qPCR results with two replicates. The captured small mammals were hosts to 16,452 individual ticks in total, of which 13,230 (80%) individual ticks from 498 hosts were successfully determined to species. A total of 3222 ticks (20%) were not identified to species because of tick damage and/or qPCR resource limitations. Of ticks across all life stages, 12,989 (98%) were *I. ricinus* and 241 (2%) were *I. trianguliceps*. Of all *I. trianguliceps*, 193 (80%) were larvae, 44 (18%) were nymphs, and 4 (2%) were adults. Of all *I. ricinus*, 12,428 (96%) were larvae, 559 (4%) were nymphs (Table 1), and 2 (< 1%) were adults. Larval *I. ricinus* presence was very high (96–100%) in all host species. On average, the wood mouse harboured most *I. ricinus* larvae (31 larvae per individual, SE = 2.5), followed by the bank vole (24, SE = 1.6) and the common shrew (20, SE = 2.7; Additional file 1: Table S3). A total of 30% of *I. ricinus* larvae and 55% of nymphs had successfully ingested a partial or full blood mean from their host, respectively.

**Fig. 3:**
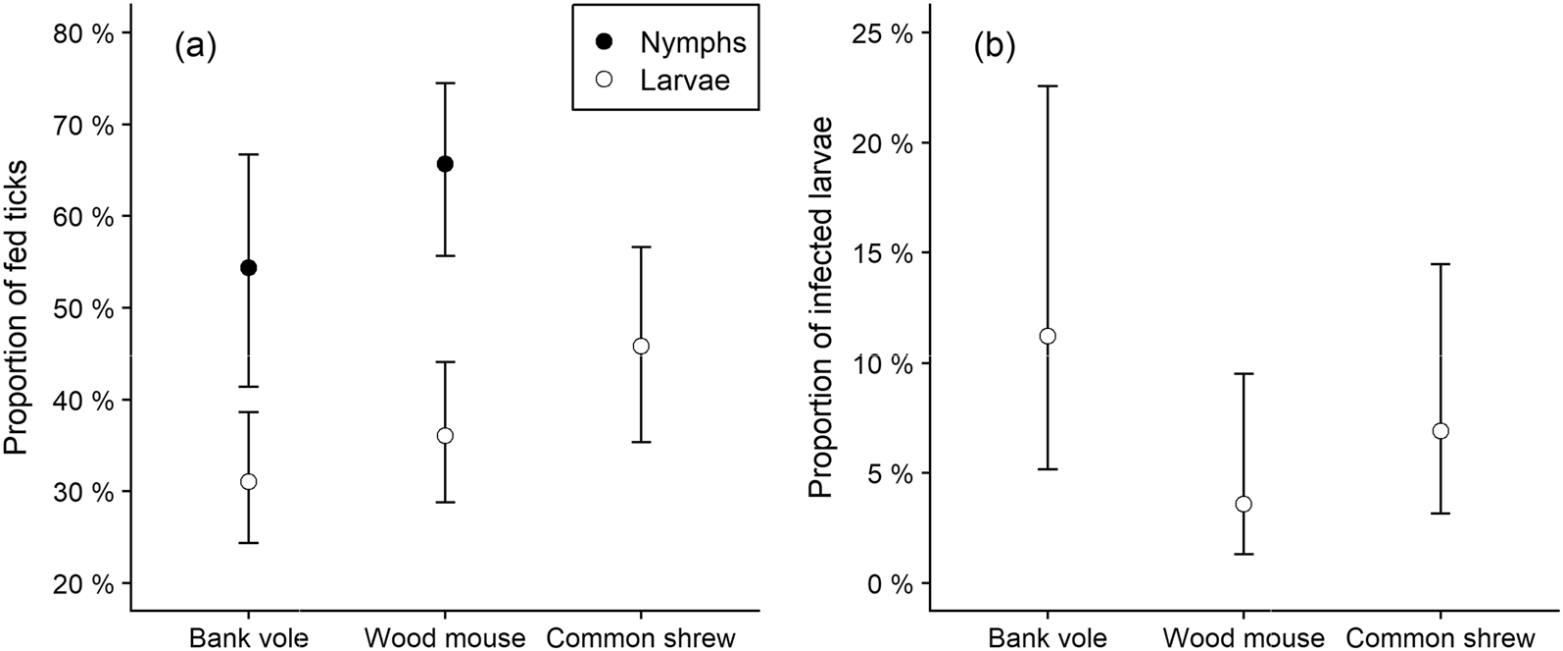
Predicted **a** proportion of successfully fed *Ixodes ricinus* larvae and nymphs on live hosts and **b** prevalence of *Borrelia burgdorferi* s.l. in blood-fed *I. ricinus* larvae from bank voles, wood mice, and common shrews captured in southeast Norway (2018–2022). Error bars denote respective 95% confidence intervals

In the models on instar tick feeding, successful blood meal ingestion was significantly influenced by host species for larvae but not for nymphs (Table 2). Both larval and nymphal feeding was influenced by whether the host was found alive for both larvae and nymphs (Table 2) but not by season (Additional file 1: Table S4). The odds of successful tick feeding on hosts found alive were 2.2 times higher for both larvae (*P* < 0.001) and nymphs (*P* = 0.006) than on hosts found dead. For larvae found on live hosts, the probability of successful blood meal ingestion was higher in common shrews (46%) compared to bank voles (31%), while levels in the wood mice (36%) were not significantly different from the bank voles (Fig. 3a). For nymphs found on live hosts, the probability of successful blood meal ingestion tended to be higher on wood mice (66%) compared to on bank voles (54%; Fig. 3a), albeit not quite significantly (*P* = 0.093, Table 2).

**Table 2:** Estimates of parameters in generalized linear mixed models on successful feeding in *Ixodes ricinus* larvae and nymphs and *Borrelia burgdorferi* s.l. infection in *I. ricinus* larvae on small mammals captured in southeast Norway (2018–2022)

Parameter	Estimate	Std. error	*z*	*P*
Larval feeding	(logit-link)			
Intercept^a^	−1.578	0.167	−9.443	< 0.001
sp = common shrew	0.628	0.163	3.849	< *0.001*
sp = wood mouse	0.227	0.131	1.734	0.083
Host status = live	0.780	0.123	6.358	< *0.001*
Nymphal feeding	(logit-link)			
Intercept^a^	−0.594	0.236	−2.516	0.012
sp = wood mouse	0.473	0.282	1.679	0.093
Host status = live	0.768	0.280	2.740	*0.006*
Larval infection	(logit-link)			
Intercept^b^	−3.118	0.494	−6.307	< 0.001
sp = common shrew	−0.530	0.521	−1.016	0.309
sp = wood mouse	−1.221	0.607	−2.013	*0.044*
Tick fed = true	1.047	0.355	2.952	*0.003*

Significant *P*-values are highlighted in italics

^a^Corresponds to bank vole found dead

^b^Corresponds to unfed larva on a bank vole

The analysis of infected *I. ricinus* larvae from hosts with confirmed *B. burgdorferi* s.l. infections included a total sample of 781 larvae collected from 58 hosts with positive *B. burgdorferi* s.l. infection. The infected hosts included 21 bank voles, 20 common shrews, and 17 wood mice. Of the 781 larvae, 53 (7%) were infected with *B. burgdorferi* s.l., of which 25 were from bank voles, 22 from common shrews, and 6 from wood mice (Table 3).

**Table 3:** Number of *Ixodes ricinus* larvae analysed for the presence of *Borrelia burgdorferi* s.l. The larvae were collected from small mammals captured in southeastern Norway (2018–2020) with confirmed *B. burgdorferi* s.l. infections

	Number of hosts	*I. ricinus* larvae		
		Positive	Negative	Sum
*Apodemus sylvaticus*	17	6 (3%)	223	229
*Myodes glareolus*	21	25 (12%)	178	203
*Sorex araneus*	20	22 (6%)	327	349
Sum	58	53 (7%)	728	781

In the model on *B. burgdorferi* s.l. prevalence in *I. ricinus* larvae, infection was influenced by tick fed status and host species (Table 2) but not by season (Additional file 1: Table S4). The odds of fed and partially fed larvae to be infected was 2.8 times (*P* = 0.003) higher than for unfed larvae. Fed larvae found on bank voles were more likely to be infected (11%) compared to wood mice (4%), while infection levels in ticks on common shrews (7%) were not significantly different from wood mice (4%; Fig. 3b, Table 2).

**Fig. 4:**
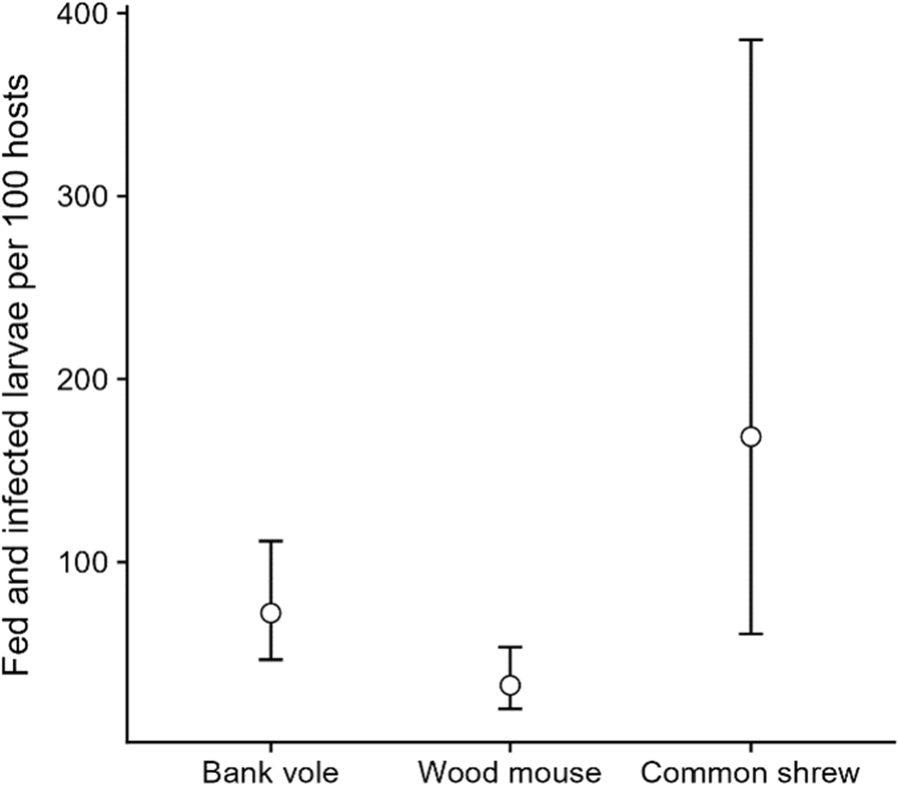
The mean number of fed *Ixodes ricinus* larvae infected with *Borrelia burgdorferi* s.l. from bank voles, wood mice, and common shrews captured in southeast Norway (2018–2022) as a function of host infection, larval burden, larval feeding on a live host, and larval infection. The number of fed and infected larvae was calculated for an equal host species abundance of 100 individuals per species. The error bars denote the interquartile range

According to our calculation of ${\text{FI}}_{{l}_{i}}$ as a function of host infection, larval burden, larval feeding on a live host, and larval infection, the estimated mean number of fed and infected *I. ricinus* larvae was highest in the common shrew (155 fed and infected larvae per 100 hosts), followed by the bank vole (70) and the wood mouse (33; Fig. 4).

## Discussion

Many pathogens and parasites have the ability to infect multiple host species and determining the host diversity is critical to understanding the epidemiology. The transmission cycle of tick-borne pathogens depends on a competent vertebrate host and a competent tick to transmit the pathogen to new hosts. Establishing host and vector competence requires both detecting pathogen transmission from an infectious tick bite to a naïve host, a lasting infection in the host, and later transmission from the infected host to feeding ticks [42]. We quantitatively estimated two principal components of this intricate interplay in the small mammal part of the Lyme disease enzootic cycle in northern Europe. We demonstrate that the feeding success of *I. ricinus* larvae and nymphs and successful host-to-larva transmission of *B. burgdorferi* s.l. differ across known small mammal host species. Furthermore, we found that a host species' ability to feed a large proportion of attached larvae did not correspond to a large proportion of infected larvae.

### Towards a quantitative understanding of the host diversity of *I. ricinus*

The host selection of *I. ricinus* per life stage is well described in many ecosystems in Europe [9, 12, 20] but typically by counting attached ticks rather than by quantifying successfully fed ticks. Few studies have quantitatively compared feeding success on small mammals. However, it has been experimentally documented that bank voles acquire resistance to *I. ricinus* after repeated tick infestations, resulting in fewer engorged ticks [4]. A previous study in the Netherlands also documented a heavier body mass of engorged larvae on wood mice compared to bank voles due to differences in blood ingestion [43]. In an experimental study in Sweden, more larvae were engorged on wood mice than bank voles because of differences in tick behaviour on the body of different host species [44]. Our study is consistent with this, showing a higher proportion of fed nymphs and larvae on wood mouse compared to bank voles. Furthermore, we found an even higher proportion of fed larval ticks on the common shrew, but few nymphs. We found a larger proportion of fed larvae and nymphs on live hosts compared to dead hosts. The mechanisms behind fewer fed ticks on dead hosts remain unknown. We did not investigate possible variation in moulting success [45].

The suitability of a given vertebrate host to ticks can depend on both body size and shape, skin thickness, immune response, and grooming behaviour [24, 46]. Red fox (*Vulpes vulpes*) appears to have immunological defences against ticks as many dead *I. ricinus* ticks are found incapsulated in the subcutaneous tissue [25–29]. For *Ixodes scapularis*, the main vector of Lyme disease in North America, some medium-sized mammals kill 83–96% of larval ticks that attempt to attach and feed [24]. However, it remains unknown whether tick grooming behaviour is present in different European small mammalian hosts [47]. An experimental study found marked differences in the likelihood of engorgement of larval *I. ricinus* when introduced on bank vole, wood mouse, and common shrew, and it was suggested that active host selection of ticks may partly explain differences in feeding success across hosts [44].

### Successful host-to-larva transmission of the pathogen

About half of pathogenic viruses and bacteria are generalists with the ability to infect more than one host [42]. The host diversity of the pathogens causing Lyme disease is typically more restricted than for the tick, with the different genospecies of *B. burgdorferi* s.l. being adapted to different groups of vertebrate hosts [9, 48]. In Europe, the competence of *I. ricinus* to vector various genospecies of *B. burgdorferi* s.l. is well documented [20, 49], but the understanding of the host diversity is mainly qualitative. As expected, we found that fed and partially fed larvae were 2.8 times more likely to be infected than unfed larvae, since host-to-tick transfer of *B. burgdorferi* s.l. from host to feeding tick is known to take several hours [50]. Among fed larvae, those from bank voles were more often infected (11%) than those from common shrews (7%) and wood mice (4%).

Whether sympatric species of competent small mammal hosts differ in their ability to pass infection to feeding ticks has mainly been investigated under laboratory conditions [51–55], but results are largely consistent with our field observations. An experimental study found increased levels of *B. burgdorferi*-specific antibodies and a corresponding decrease in host-to-tick transmission levels in both the wood mouse and yellow-necked mouse compared to the bank vole [7]. The efficiency of host-to-tick transmission varied markedly between the five individuals of wood mouse and yellow-necked mouse (*Apodemus flavicollis*) in an experimental study [55], but inference about species and individual variation in transmission was difficult because of the small sample size [55]. In Sweden, *B. burgdorferi* s.l. spirochaete abundance varied in different tissues between infected yellow-necked mouse, bank vole and common shrew [56], which may explain some of the between-species variation in host-to-tick transmission efficiency.

There is considerable variation in *B. afzelii* strains in Europe [57], and strain specificity can influence transmission in experimental settings [58, 59]. The consistent patterns of host-to-tick transmission across host species in experimental and field studies with *B. afzelii* from different regions may suggest that strain variation influence is comparably less important. However, it is premature to conclude about the quantitative role of strain variation in larger regions without further studies.

### Species composition of the host community

The relative importance of different vertebrates to *I. ricinus* ticks may vary across Europe depending on differences in abundance and in species composition [10]. The yellow-necked mouse is a common host of *I. ricinus* on the European continent [60] but is less abundant in northern Europe [33]. Consistent weekly observations of *I. ricinus* have been reported as far north as 68°N in Norway [61], where even the wood mouse is uncommon [62]. Although often neglected, shrews are important hosts for ticks and tick-borne pathogens at northern latitudes in Europe [18, 33, 63], but reportedly less so in southern Europe [64, 65]. Voles and shrews are likely commonly encountered small mammalian hosts in the northernmost range limits of *I. ricinus* [19, 63].

The contribution of different host species to the enzootic cycle of Lyme disease ultimately depends on both host density and realized reservoir competence [51]. The realized host reservoir competence can be defined as the number of blood-fed larvae that become infected with *B. burgdorferi* s.l. and later moult to nymphs [51, 66], which is a function of host infection, larval tick burden, successful larval feeding, host-to-larva transmission, and moulting success [53]. Here, we quantitatively confirmed that the infected bank vole, but also the common shrew, transmits *B. burgdorferi* s.l. to a higher proportion of feeding *I. ricinus* larvae compared to the wood mouse. Importantly, the between-species variation in infected larvae was greater than the corresponding variation in blood-fed larvae. The lower proportion of fed larvae on the bank vole and the common shrew was exceeded by the higher proportion of infected larvae, resulting in a relatively higher production of fed and infected larvae in the common shrew and bank vole compared to the wood mouse. Note that this is given the assumption of constant relative species variation in feeding and host-to-tick transmission at varying host density. However, our results highlight the common shrew and bank vole as key hosts in producing fed *I. ricinus* larvae infected with *B. burgdorferi* s.l. at northern latitudes.

## Conclusions

Many pathogens and parasites can infect multiple host species, and we provide a new level of detail for the quantitative differences in host and vector competence critical for understanding the epidemiology of Lyme disease in northern Europe. Common small mammals form a particularly important component of the enzootic cycle of Lyme disease in Europe because of their dual role in feeding larval *I. ricinus* ticks and harbouring the most common genospecies of *B. burgdorferi* s.l. We add a further level of detail by highlighting variation in principal components of *I. ricinus* vector competence and the realized host competence between small mammalian species depending on their contribution to successfully feeding and infecting ticks. Another important transmission cycle affecting Lyme disease risk in Europe is the circulation of *B. garinii* in birds [67–69], and similar studies of instar stages of *I. ricinus* feeding on important host species of birds would enable a more complete understanding of mechanisms behind Lyme disease emergence in humans.

### Supplementary Information

**Additional file 1: Table S1.** An overview of samples sizes of captured small mammals with live and lethal traps per season between 2018 and 2022 in Son, Viken County, Norway. **Table S2.** Sequences and adjusted primer set and probe concentrations in respective multiplex real-time quantitative PCR assays for detecting (A) *Borrelia burgdorferi* s.l. and *Anaplasma phagocytophilum* and (B) *Ixodes ricinus* and *I. trianguliceps*. **Table S3.** Population level mean and median and interquartile range (IQR) of larval *Ixodes ricinus* tick burden on individual hosts, proportion of hosts infected with *Borrelia burgdorferi* s.l., mean proportion of fed *I. ricinus* larvae, and mean proportion of fed and *B. burgdorferi* s.l. infected *I. ricinus* larvae on captured small mammals between 2018 and 2022 in Son, Viken County, Norway. **Table S4.** Estimates of parameters in generalized linear mixed models on successful feeding in *Ixodes ricinus* larvae and nymphs and *Borrelia burgdorferi* s.l. infection in *I. ricinus* larvae on small mammals captured in southeast Norway (2018–2022) as a function of season and whether the host was found dead or alive.

## Data Availability

The data supporting the findings of the study must be available within the article and/or its supplementary materials, or deposited in a publicly available database.
