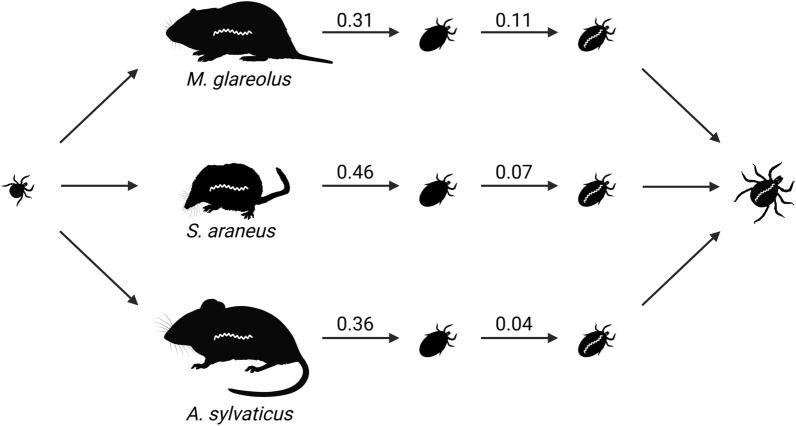# Correction: Vector competence of *Ixodes ricinus* instars for the transmission of *Borrelia burgdorferi* sensu lato in different small mammalian hosts

**DOI:** 10.1186/s13071-024-06249-x

**Published:** 2024-04-02

**Authors:** Lars K. Lindsø, Hildegunn Viljugrein, Atle Mysterud

**Affiliations:** 1 Centre for Ecological and Evolutionary Synthesis (CEES), Department of Biosciences, University of Oslo, Blindern, P.O. Box 1066, 0316 Oslo, Norway; 2 Norwegian Veterinary Institute, P.O. Box 64, 1431 Ås, Norway; 3 Norwegian Institute for Nature Research (NINA), Torgarden, P.O. Box 5685, 7485 Trondheim, Norway

**Correction: Parasites & Vectors (2024) 17:23** 10.1186/s13071-023-06110-7

Following publication of the original article [1], it came to the authors’ attention that results regarding larval *Ixodes ricinus* feeding success in wood mice (“*Apodemus sylvaticus*, 36%”) and bank voles (“*Myodes glareolus*, 31%”) had been interchanged in the abstract and graphical abstract of the article. The article has since been corrected. The authors thank you for reading this erratum and apologize for any inconvenience caused.