# Supplementary material for: Vector competence of Ixodes ricinus instars for the transmission of Borrelia burgdorferi sensu lato in different small mammalian hosts
Source: Parasit Vectors. 2024 Jan 18;17:23. doi: 10.1186/s13071-023-06110-7 (PMC10797980; doi:10.1186/s13071-023-06110-7)
Supplement: Supplementary file 1 — Additional file 1: Table S1. An overview of samples sizes of captured small mammals with live and lethal traps per season between 2018 and 2022 in Son, Viken County, Norway. Table S2. Sequences and adjusted primer set and probe concentrations in respective multiplex real-time quantitative PCR assays for detecting (A) Borrelia burgdorferi s.l. and Anaplasma phagocytophilum and (B) Ixodes ricinus and I. trianguliceps. Table S3. Population level mean and median and interquartile range (IQR) of larval Ixodes ricinus tick burden on individual hosts, proportion of hosts infected with Borrelia burgdorferi s.l., mean proportion of fed I. ricinus larvae, and mean proportion of fed and B. burgdorferi s.l. infected I. ricinus larvae on captured small mammals between 2018 and 2022 in Son, Viken County, Norway. Table S4. Estimates of parameters in generalized linear mixed models on successful feeding in Ixodes ricinus larvae and nymphs and Borrelia burgdorferi s.l. infection in I. ricinus larvae on small mammals captured in southeast Norway (2018–2022) as a function of season and whether the host was found dead or alive. [file 13071_2023_6110_MOESM1_ESM.pdf]

Supporting information

Vector competence of *Ixodes ricinus* instars for the transmission of *Borrelia burgdorferi* sensu lato on different small mammalian hosts

Lars K. Lindsø, Hildegunn Viljugrein, and Atle Mysterud

Table S1: An overview of samples sizes of captured small mammals with live and lethal traps per season between 2018 and 2022 in Son, Viken county, Norway.

		2018		2019		2020		2021		2022	
	Sum	Spring	Fall	Spring	Fall	Spring	Fall	Spring	Fall	Spring	Fall
% Live traps		100	80	80	80	48	48	48	48	0	0
A. sylvaticus	156	2	9	20	16	27	30	23	11	11	7
M. agrestis	14	1	0	0	3	0	9	0	0	0	1
M. glareolus	258	3	16	4	12	10	143	10	27	10	23
S. araneus	106	2	27	1	21	1	36	1	14	0	3
S. minutus	23	0	3	0	5	3	11	0	1	0	0
Sum	557	8	55	25	57	41	229	34	53	21	34

Table S2: Sequences and adjusted primer set and probe concentrations in respective multiplex real-time quantitative PCR assays for detecting A) *Borrelia burgdorferi* s.l. and *Anaplasma phagocytophilum* and B) *I. ricinus* and *I. trianguliceps*, with respective target genes, product size (bp), sequences, and concentration (µM). Infection prevalence of *A. phagocytophilum* was not considered in the present study.

A) Infection prevalence	**Target**	**bp**	**Sequence**	**µM**
A. phagocytophilum	16S rRNA	75		
F primer			ATGGAAGGTAGTGTTGGTTATGGTATT	0.900
R primer			TTGGTCTTGAAGCGCTCGTA	0.900
Probe			[HEX]TGGTGCCAGGGTTGAGCTTGAGATTG[TAMRA]	0.125
B. burgdorferi s.l.	23S rRNA	77		
F primer			CGAGTCTTAAAAGGGCGATTTAGT	0.700
R primer			GCTTCAGCCTGGCCATAAATAG	0.700
Probe			[6FAM]AGATGTGGTAGACCCGAAGCCGAGTG[TAMRA]	0.175
B) Tick species				
I. ricinus	*ITS2*	72		
F primer			TTGACTGTGTCGGATCGTGG	0.600
R primer			TCGCTCCGAGAACGTGAAAA	0.600
Probe			[5TexRd-XN]ACGCATGTTTATGCTTTCTTGCGTTGCG[Iowa black]	0.200
I. trianguliceps	*ITS2*	128		
F primer			CGCGAACGTTGGAATCGTAC	0.600
R primer			GGCTCCAGTATTCATCGGGG	0.600
Probe			[5HEX]CCGTGCGTCGTAGCCATCCGT[3IAbRQSp]	0.200

Table S3: Population level mean and median and interquartile range (IQR) of larval *Ixodes ricinus* tick burden on individual hosts, proportion of hosts infected with *B. burgdorferi* s.l., mean proportion of fed *I. ricinus* larvae, and mean proportion of fed and *B. burgdorferi* s.l. infected *I. ricinus* larvae on captured small mammals between 2018 and 2022 in Son, Viken county, Norway. SE denotes the standard error of the sample proportion for host infection, and model (logit) scale standard errors for larval feeding and larval infection, respectively.

	**Larval burden**			**Host infection**		**Larval feeding**		**Larval infection**	
	Mean	Median	IQR	%	SE	Mean	SE	Mean	SE
Apodemus sylvaticus	31	19	11-39	0.30	0.04	0.36	0.17	0.04	0.53
Myodes glareolus	24	16	9-31	0.40	0.03	0.31	0.17	0.11	0.43
Sorex araneus	20	13	6-20	0.51	0.05	0.46	0.22	0.07	0.42

Table S4: Estimates of parameters in generalized linear mixed models on successful feeding in *I. ricinus* larvae and nymphs, and *B. burgdorferi* s.l. infection in *I. ricinus* larvae, on small mammals captured in South-East Norway (2018-2022) as a function of season and whether the host was found dead or alive. Significant *P*-values are italicized. Random intercepts of "trap station", "host ID" and "year" were included. The intra-class correlation (ICC) of the random intercepts were calculated by the R-package performance version 0.10.4.

**Parameter**	**Estimate**	**Std. error**	***z***	***P***
Larval feeding [2]	(logit-link)			
Intercept [1]	-1.197	0.206	-5.800	<0.001
Season = Spring	-0.087	0.127	-0.684	0.494
Status = Live	0.666	0.118	5.650	*<0.001*
Nymphal feeding [3]	(logit-link)			
Intercept [1]	-0.449	0.392	-1.146	0.252
Season = Spring	0.323	0.342	0.945	0.345
Status = Live	0.772	0.373	2.068	*0.039*
Larval infection [4]	(logit-link)			
Intercept [1]	-3.144	0.379	-8.292	<0.001
Season = Spring	-0.336	0.574	-0.585	0.559
Status = Live	0.771	0.530	1.455	0.146

[1]: The intercept corresponds to a host found dead in fall.

[2]: The ICC of the random intercept "host ID" = 0.202 and "year" = 0.040. The random intercept variance for "trap station" was close to zero.

[3]: The ICC of the random intercept "host ID" = 0.156, "year" = 0.016, and "trap station" = 0.007.

[4]: The ICC of the random intercept "trap station" = 0.274. The random intercept variance for "host ID" and "year" were close to zero.
